# The Postoperative Effect of Sugammadex versus Acetylcholinesterase Inhibitors in Colorectal Surgery: An Updated Meta-Analysis

**DOI:** 10.3390/jcm12093235

**Published:** 2023-04-30

**Authors:** Sascha Vaghiri, Dimitrios Prassas, Sarah Krieg, Wolfram Trudo Knoefel, Andreas Krieg

**Affiliations:** 1 Department of Surgery (A), Heinrich-Heine-University and University Hospital Duesseldorf, Moorenstr. 5, Bldg. 12.46, 40225 Duesseldorf, Germany; sascha.vaghiri@med.uni-duesseldorf.de (S.V.); dimitrios.prassas@med.uni-duesseldorf.de (D.P.);; 2 Clinic for Gastroenterology, Hepatology and Infectious Diseases, Heinrich-Heine-University and University Hospital Duesseldorf, 40225 Duesseldorf, Germany; sarah.krieg@med.uni-duesseldorf.de

**Keywords:** sugammadex, acetylcholinesterase inhibitors, colorectal surgery, operative outcome, gastrointestinal motility

## Abstract

Background: the aim of this meta-analysis was to evaluate the postoperative effects of neuromuscular blockade reversal with sugammadex compared with acetylcholinesterase inhibitors in colorectal surgery. Methods: A systematic literature search was performed for studies comparing the postoperative course of patients receiving neuromuscular blockade reversal with either sugammadex or acetylcholinesterase inhibitors (control) after colorectal surgery. Data from eligible studies were extracted, qualitatively assessed, and included in a meta-analysis. Odds ratios and standardized mean differences with 95% confidence intervals (CIs) were calculated. Results: Five studies with a total of 1969 patients were included (sugammadex n = 1137, control n = 832). Sugammadex reversal resulted in a significantly faster return of defecation or flatus after surgery compared to acetylcholinesterase inhibitors (SMD 13.01, 95% CI 6.55–19.46, *p* = < 0.0001). There were no significant differences between the two groups in other clinical outcomes such as surgical morbidity and length of hospital stay. Conclusion: The present data support the beneficial impact of sugammadex on gastrointestinal motility after colorectal surgery. However, the effect of sugammadex on the prevention of surgical complications and a prolonged hospital stay is diminishing. Larger randomized controlled trials with standardized study protocols are needed to validate the results presented here.

## 1. Introduction

Postoperative ileus (POI) is unfortunately a common phenomenon after gastrointestinal surgery and contributes to high morbidity, prolonged hospital stay, increased readmission rates, and thus high hospital costs [1,2]. Even in the era of improved recovery programs, POI rates between 15% and 30% are reported after colorectal surgery [3,4]. Nowadays, muscle relaxation with neuromuscular blocking agents (NMBAs) is widely used to improve surgical conditions and reduce the rate of postoperative adverse events [5,6]. Residual effects of neuromuscular blockade and muscle weakness are associated with postoperative pulmonary complications (POPCs) and impaired clinical recovery in the postanesthesia care unit (PACU) [7,8]. Acetylcholinesterase inhibitors such as neostigmine or pyridostigmine have traditionally been used to reverse the effects of NMBAs [9]. Potential cholinergic side effects of acetylcholinesterase inhibitors due to peripheral muscarinic activation are treated via the concomitant administration of anticholinergics (e.g., atropine or glycopyrrolate) [10]. Sugammadex is a modified γ-cyclodextrin that forms a stable 1:1 complex with aminosteroidal neuromuscular blockers such as rocuronium or vecuronium, reducing their availability to nicotinic receptors [11]. In a Cochrane-based meta-analysis of 41 included studies, sugammadex was found to have a faster potential to antagonize the rocuronium-induced neuromuscular blockade (regardless of the depth of blockade) and 40% fewer adverse events compared with neostigmine [12]. However, there are conflicting results in the literature regarding postoperative defecation when sugammadex or neostigmine is administered as their effect on bowel motility and recovery has still not been fully elaborated upon [13,14]. This could be partly related to the fact that no unified and reproducible outcome measures of gastrointestinal motility function throughout the literature exists [15]. Therefore, our primary objective was to critically evaluate the role of sugammadex compared to “classical” acetylcholinesterase inhibitors in the postoperative course of patients undergoing colorectal surgery by means of a systematic review and meta-analysis. Special attention will be paid to all reported parameters of postoperative gastrointestinal motility.

## 2. Material and Methods

Prior to the study initiation, the study protocol was registered in the International Prospective Register of Systematic Reviews (PROSPERO CRD 42022383245). This meta-analysis was conducted in accordance with the current PRISMA statement (Preferred Reporting Items for Systematic Reviews and Meta-Analyses) [16] and the latest version of the Cochrane Handbook for Systematic Reviews of Interventions [17].

### 2.1. Eligibility Criteria and Group Definition

All studies comparing postoperative clinical outcomes after the reversal of neuromuscular blockade with sugammadex or an acetylcholinesterase inhibitor (defined as the comparator) in patients undergoing colorectal surgery were included. To avoid heterogeneity, only studies in which 100% of patients underwent colorectal surgery for any reason were included. Particular attention was paid to postoperative gastrointestinal motility parameters such as ileus, time to first bowel movement or flatus, and time to first solid food intake. Other outcomes of interest included postoperative morbidity and mortality, number of pulmonary events, length of postoperative hospital stay, and rate of readmission to the hospital or intensive care unit. To be included in the analysis, studies had to report at least one of the outcomes listed above. Publications that were randomized controlled trials (RCTs) or prospective or retrospective comparative cohort studies were included in the analysis. Disagreements or differing conclusions in the selection of studies were resolved either via consensus or consultation with an independent third author (S.K.).

### 2.2. Literature Search

A systematic electronic search of the Pubmed (Medline) and Scopus databases without time or language restrictions was performed to identify articles comparing outcomes in patients with colorectal surgery after the reversal of neuromuscular blockade with sugammadex or an acetylcholinesterase inhibitor. The following search terms were used in combination with the Boolean operators AND or OR: “sugammadex”, “Bridion^®^”, “neuromuscular reversal”, “neostigmine”, “acetylcholine”, “pyridostigmine”, and “colorectal”. In addition, the reference list of retrieved articles (including systematic reviews, case reports, editorials, or experimental studies) was reviewed to identify potentially relevant citations for analysis. Two reviewers (S.V. and D.P.) performed the primary search and independently assessed each abstract and eligible study for relevance for inclusion in the meta-analysis. A third reviewer (S.K.) was consulted as needed. The final literature search was performed on 20 January 2023.

### 2.3. Data Extraction and Outcome Measures

Two authors (S.V. and D.P.) independently recorded all available and relevant data from studies meeting the inclusion criteria on a self-generated electronic data extraction sheet. Study- and patient-specific information included country of origin, year of publication, study design, exclusion criteria, enrollment period, type and composition of neuromuscular reversal drug, number of patients enrolled per group and their demographics (age, sex, body mass index (BMI), ASA class, and comorbidities), surgical indication, proportion of laparoscopic and open surgery procedures, duration of anesthesia (min), and follow-up period. The primary endpoint was time (hours) to first documented postoperative bowel movement or flatus. The secondary postoperative endpoints analyzed were ileus (as individually defined by the authors), time to first solid oral intake (hours), postoperative nausea and vomiting (PONV), reported adverse pulmonary events (pooled composite of pneumonia, hypoxemia, postoperative supplemental oxygen, acute respiratory distress syndrome, pulmonary embolism, and emergency sugammadex use), urinary tract infection, anastomotic leak, bleeding, postoperative wound infection, length of postoperative hospital stay (days), PACU stay (minutes), reoperation rate (within 30 days), hospital and ICU readmission rate, and mortality. Again, discrepancies in data extraction were resolved via consensus or reassessment by an independent third author (S.K.).

### 2.4. Quality Assessment

The quality of the included non-randomized trials was independently assessed by the authors using the ROBINS-I tool [18], which covers seven different domains of bias at three time points in each trial: before intervention (confounding and selection of participants), during intervention (classification of interventions), and after intervention (bias due to deviations from planned interventions, missing data, measurement of outcomes, and selection of reported outcomes). Based on these domains, a final assessment of the overall risk of bias for each included study was possible in the categories “low risk”, “moderate risk”, “high risk”, and “critical risk”. The Grading of Recommendations, Assessment, Development, and Evaluation (GRADE) method [19] with four assigned levels of evidence (high, moderate, low, and very low) was used to adequately document the strength of evidence for the significant outcomes [19,20].

### 2.5. Statistical Analyses

Statistical analysis was performed using RevMan software (version 5.3; Copenhagen: The Nordic Cochrane Centre, The Cochrane Collaboration, 2014). Pairwise meta-analyses were performed. For each endpoint of interest, summary treatment effect estimates with 95% confidence intervals (CIs) were calculated. For dichotomous endpoints, the odds ratio (OR) was chosen as the effect measure. For continuous outcomes, standardized mean differences (SMDs) were calculated. The methods proposed by Luo et al. [21] and Wan et al. [22] were used to calculate the mean and standard deviation (SD) from the available median and interquartile range data. The degree of heterogeneity among the included studies was interpreted as follows after applying the Cochrane Q test (chi-square test; Chi^2^) and measuring inconsistency (I^2^): 0–30% low heterogeneity, 30–50% moderate heterogeneity, and 50–90% substantial heterogeneity [17,23]. If heterogeneity was low or moderate (I^2^ < 50%), summary estimates were calculated using a fixed-effects method. Where appropriate, subgroup analyses were performed to examine heterogeneity in the results. Publication bias tests and funnel plots were not performed due to the small number of studies included in the meta-analysis, as recommended [17].

## 3. Results

### 3.1. Study and Patient Characteristics

**Figure 1:**
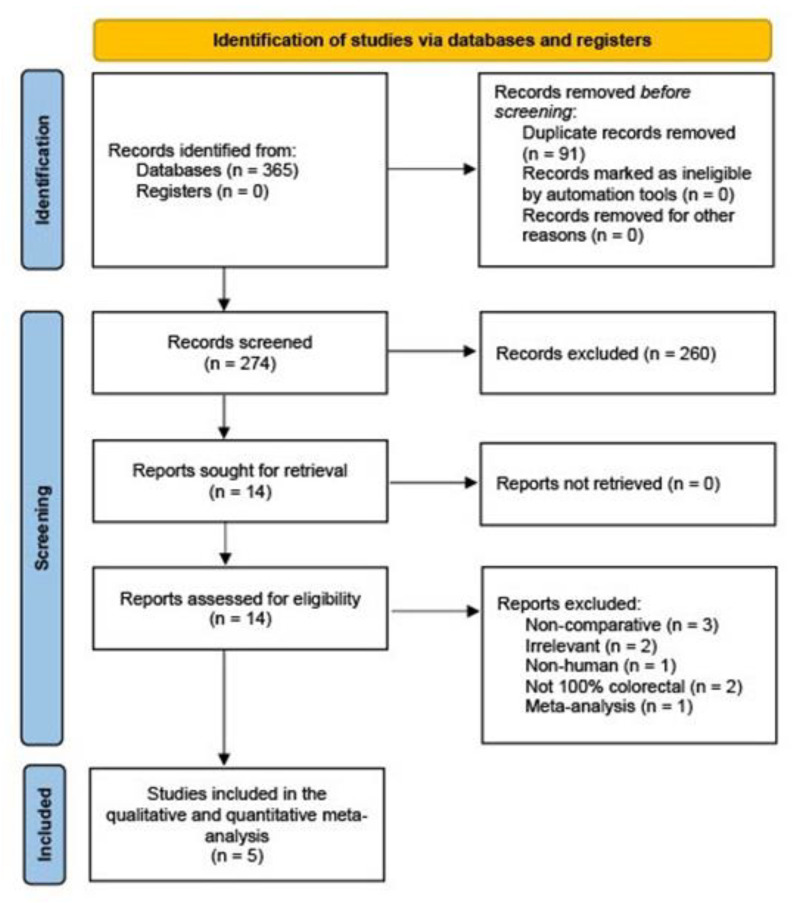
Flowchart for identifying and selecting studies for review analysis.

The initial database search using the predefined keywords yielded 365 potentially relevant abstracts. Of these, 14 full-text articles were screened for eligibility and finally 5 non-randomized observational studies (1 multi-center prospective and 4 single-center retrospective) comparing the outcome of neuromuscular blockade reversal with sugammadex and acetylcholinesterase inhibitors were included in the qualitative and quantitative data analysis [24,25,26,27,28]. The PRISMA flowchart for the literature search is shown in Figure 1. Of the total 1969 patients included (1114 men/855 women), 1137 were assigned to the sugammadex group and 832 were assigned to the acetylcholinesterase inhibitor group (control). Two studies used pyridostigmine as the acetylcholinesterase inhibitor [24,26], and three studies used neostigmine [25,27,28]. Three studies included both malignant and non-malignant cases [25,27,28], while two studies included only patients with colorectal cancer [24,26]. The rate of laparoscopic surgery ranged from 20–100% in the sugammadex group to 22–100% in the control group. Inpatient [25,26] and up to 30 days [24,27,28] follow-up data were presented. The study by Serrano et al. [27] was a prespecified substudy of the POWER trial [29]. Table 1, Table 2 and Table 3 provide a detailed summary of the study and clinical characteristics.

### 3.2. Study Quality and Risk of Bias

**Figure 2:**
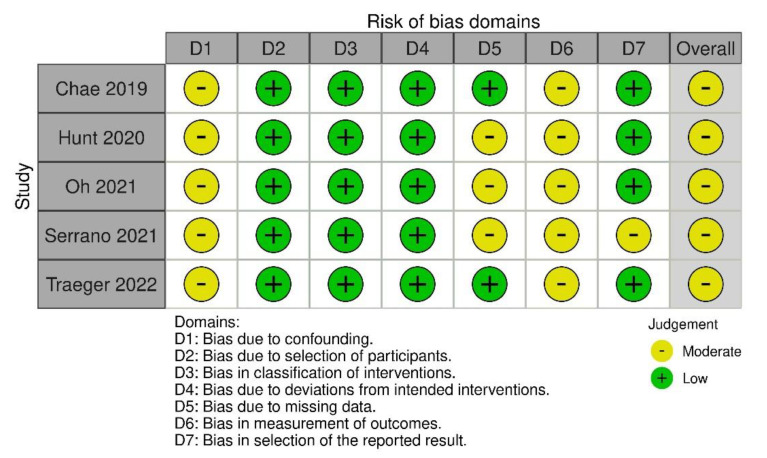

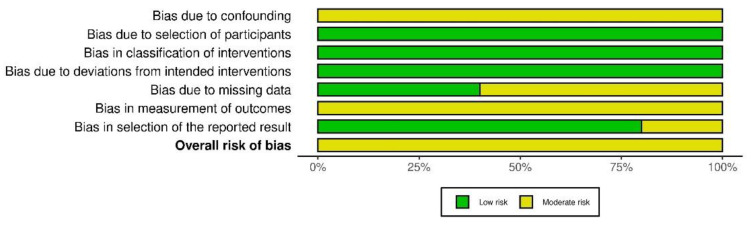
Summary of risk of bias and graphical representation of included studies [24,25,26,27,28] based on the ROBINS-I tool.

The risk of bias (Figure 2) was moderate in all included studies according to the Robins I tool [18]. However, the most important limiting factor in terms of bias was the non-randomized and observational study design of all of the studies. In addition, the definition of gastrointestinal motility outcomes varied widely among the included studies, and not all outcomes of interest were available in each study. Based on the GRADE method [19], the level of evidence for the primary endpoint was rated as being very low (Table 4).

### 3.3. Gastrointestinal Motility Outcomes

#### 3.3.1. Time to First Bowel Movement or Flatus

**Figure 3:**
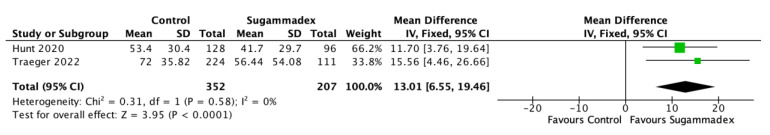
Forest plot comparing time to first bowel movement or flatus between sugammadex-reversed cases and controls from the studies published by Hunt et al. [25] and Traeger et al. [28].

Time to first bowel movement or flatus as the primary endpoint was reported in two studies with 559 patients [25,28]. In the sugammadex group, the time to first postoperative bowel movement or flatus was significantly shorter than in the control group (SMD 13.01, 95% CI 6.55–19.46, *p* < 0.0001). Importantly, heterogeneity was low (I^2^ = 0%, Chi2 test: *p* = 0.58) (Figure 3).

#### 3.3.2. Time to First Oral Diet Intake

**Figure 4:**
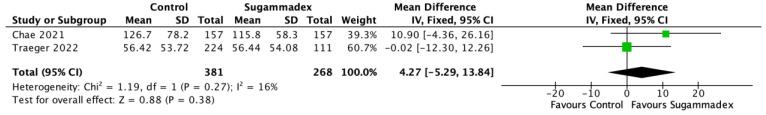
Forest plot comparing time to first oral food intake between sugammadex and control from the studies published by Chae et al. [24] and Traeger et al. [28].

Two studies reported on the time to first oral food intake [24,28]. Meta-analysis revealed no statistically significant difference between the two groups regarding the time to first postoperative oral food intake (SMD 4.27, 95% CI −5.29–13.84, *p* = 0.38). The degree of heterogeneity was low (I^2^ = 16%, Chi2 test: *p* = 0.27) (Figure 4).

#### 3.3.3. Ileus

**Figure 5:**
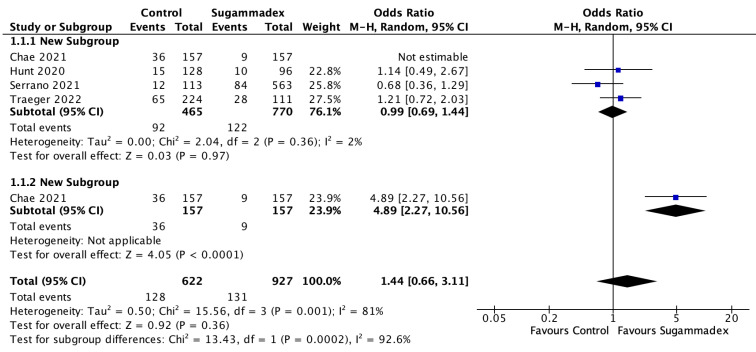
Forest plot comparing ileus rates in sugammadex-antagonized cases and controls from the studies published by Chae et al. [24], Hunt et al. [25] , Serrano et al. [27] and Traeger et al. [28].

Postoperative ileus was reported in four studies [24,25,27,28]. A meta-analysis of the pooled data showed no significant difference between the sugammadex and acetylcholinesterase inhibitor groups in terms of postoperative ileus rate (OR 1.44, 95% CI 0.66–3.11, *p* = 0.36). The data for this outcome were highly heterogeneous (I^2^ = 81%, Chi^2^-test: *p* = 0.001). The source of heterogeneity was identified in the study by Chae et al. [24]. However, the subsequent subgroup with low heterogeneity (I^2^ = 2%, Chi2 test: *p* = 0.36) still demonstrated no difference in the reported ileus rates between both groups (OR 0.99, 95% CI 0.69–1.44, *p* = 0.97) (Figure 5).

### 3.4. Non-Gastrointestinal Motility Outcomes

Analysis of secondary endpoints other than gastrointestinal motility (urinary tract infection, pulmonary morbidity, PONV, postoperative hospitalization, anastomotic leak, bleeding, wound infection, ICU stay, repeat surgery, repeat hospitalization, repeat ICU stay, and mortality) showed no statistically significant difference between the sugammadex and control groups. A detailed summary is shown in Table 5.

## 4. Discussion

Although the level of evidence seems to be very low, the results of our meta-analysis suggest that the reversal of the neuromuscular blockade with sugammadex compared with acetylcholinesterase inhibitors accelerates postoperative bowel motility in colorectal surgery. However, other surrogate markers of gastrointestinal motility such as ileus or time to first solid food intake were not affected by the type of reversal agent. In addition, no significant effect on clinical outcomes, including pulmonary events, postoperative complications and mortality, or length of hospital stay was observed in patients receiving sugammadex. To our knowledge, this is the first meta-analysis comparing sugammadex and classic acetylcholinesterase inhibitors exclusively in colorectal surgery, with a special focus on gastrointestinal motility parameters. With the increasing number of colorectal procedures, enhanced recovery after surgery (ERAS) pathways with a multimodality approach toward minimizing postoperative ileus [30] are becoming increasingly important as they have been shown to reduce postoperative complications and length of hospital stay [31].

The pharmacological effect of sugammadex lies in its ability to create a tight 1:1 complex with aminosteroid neuromuscular blocking agents. This complex results in a decrease in free NMBA plasma concentration, promoting a gradient shift from the peripheral compartment (including neuromuscular junction) into plasma [11,32]. Furthermore, the potential impact of sugammadex on gastrointestinal motility is linked to the affinity to bind with steroid hormones [28,33]. In contrast, the administration of anticholinergics to prevent the cholinergic side effects of acetylcholinesterase inhibitors may unintentionally cause adverse gastrointestinal effects such as constipation or ileus [11].

Deljou et al. [34] also demonstrated in a large cohort of transabdominal surgery patients that reversal with sugammadex was associated with a faster occurrence of the first postoperative bowel movement compared to neostigmine/glycopyrrolate, whereas the length of hospital stay showed no statistical difference between the two reversal groups. However, this retrospective study did not report the overall rate of colorectal surgery, limiting the power in this subgroup. The same results were observed in another study by Cho et al. [35], who analyzed the recovery time of gastrointestinal motility in 736 patients after open pancreaticoduodenectomy. Sugammadex administration resulted in a lower incidence of delayed flatus passage and delayed tolerance to oral food intake compared to neostigmine administration.

Time to first bowel movement was also studied in three randomized trials with conflicting results. While An et al. [36] showed that the use of sugammadex in laparoscopic cholecystectomy was associated with earlier first postoperative flatus, the studies by Claroni et al. [37] and Sen at al. [33] failed to demonstrate a significant effect of sugammadex on gastrointestinal motility in robotic cystectomy and thyroid surgery, respectively. One explanation for the incongruent and heterogeneous results regarding intestinal motility in our meta-analysis and in the literature may be the different definition of ileus. In contrast to Chae et al. [24] and Serrano et al. [27] who did not specify the definition of ileus, Hunt and co-workers [25] reported ileus rates based on clinical records and documentation, and Traeger et al. [28] defined ileus as the failure to reach GI-2 (consisting of time to defecate and oral solid food intake without nausea) after four postoperative days. Furthermore, the development of postoperative ileus is triggered by a complex neuro-immuno-inflammatory response and the μ-opioid receptor activation pathway [13,38]. Therefore, reduced surgical trauma and opioid-sparing postoperative analgesia may be more effective in ileus prophylaxis than the reversal of the neuromuscular blockade with sugammadex or acetylcholinesterase inhibitors [13]. Interestingly, it has been demonstrated that patients undergoing right-sided colectomy have a higher incidence of postoperative ileus compared to patients with left hemicolectomy [39]. The type and side of resection were reported in four included studies [25,26,27,28], but based on the provided data, a subgroup analysis was not possible for determining the reversal effect after right- and left-sided colectomy. Another important flaw is the lack of complementary information regarding time point and duration of sugammadex or acetylcholinesterase inhibitor administration, and not all studies mentioned the exact dosage of the applied reversal agents in their protocols [26,27,28], thus restricting definite conclusions and recommendations. Sugammadex was associated with a significant lower incidence of pulmonary reverse events, as demonstrated in a large multicenter cohort study [40]. However, in a recently published study by Alday et al. [7] with 126 patients undergoing major abdominal surgery including 80 colorectal cases, no difference was found in terms of pulmonary function in the sugammadex and neostigmine groups. In line with this observation, we also could not find a significant advantage of sugmmadex in preventing pulmonary morbidity in colorectal surgery with 1410 included patients.

In a recently published meta-analysis, Chen et al. [41] for the first time compared the postoperative outcomes of sugammadex with a control drug for neuromuscular reversal after colorectal surgery. The authors showed that there was no statistically significant difference between the two groups in total hospital length of stay and rates of adverse respiratory events. However, despite its novelty, this study has some major weaknesses in terms of study selection and results: (1) The authors included studies with varying proportions of colorectal procedures (63.3–100%). Although the majority of patients underwent colorectal surgery, the results were from a heterogeneous study population. (2) The included study by Piccioni et al. [42] investigated the outcome of patients treated with sugammadex versus a placebo, whereas all of the other analyzed studies compared the effect of sugammadex with classical non-selective cholinesterase inhibitors such as pyridostigmine or neostigmine. (3) The authors focused mainly on postoperative respiratory events and length of hospital stay, whereas other important pillars of the ERAS concept such as postoperative nutrition and ileus were not considered in the meta-analysis.

Based on the results presented, the validity of the recommendation for clinical practice is clearly limited, especially considering the retrospective design of four of the included studies, which may be subject to bias and misinterpretation. The quality of the data may vary within these retrospective studies, and thus unwanted variables may influence the result. Even though the studies included 100% colorectal surgical procedures, they show a noteworthy heterogeneity of study protocols and gastrointestinal motility outcome definitions. In order to address this important issue and with respect to the variable definitions, all provided bowel function outcome measures were included in the meta-analysis. Of note, the proposed composite GI-2 outcome as a validated and evidence-based measure [15] was only mentioned in one study [28].

Furthermore, the exclusion criteria of the individual studies vary considerably, not only with regard to the neuromuscular blocking agent and its combination preparation, but also with regard to the surgical indication. The proportion of open versus laparoscopic procedures and the extent of surgical resection as an important factor in the development of ileus are not evenly distributed.

In addition, the economic benefit of sugammadex remains controversial [43,44,45]. In a recent study from Taiwan, for example, despite better postoperative recovery, the benefits of sugammadex did not outweigh the higher costs compared with neostigmine [46]. Moreover, different healthcare systems and costs must be taken into account when considering the use of sugammadex. Although a newly approved drug is initially protected by a patent, once the patent expires, other companies can manufacture and sell the drug as a generic under a different name. The price will then be determined by competition and will usually fall. This scenario can of course be applied to sugammadex; so, it is likely that the introduction of generics will make sugammadex cheaper and more widely available in the future.

## 5. Conclusions

The use of sugammadex to reverse neuromuscular blockade during colorectal surgery was associated with faster postoperative defecation or flatus compared with acetylcholinesterase inhibitors. However, sugammadex did not show significant superiority in other gastrointestinal motility parameters and clinical endpoints such as length of postoperative hospital stay and complications. Due to the lack of high-quality randomized trials and varying definitions of outcome measures for the postoperative return of bowel movement, the results must be interpreted with caution and the value of sugammadex in colorectal surgery requires further investigation.

## Figures and Tables

**Table 1:** Study characteristics.

Author	Year	Origin	Study Design	Recruitment Period	Sample Size	Exclusion Criteria	Colorectal Cases (%)	Reversal agents	Follow-Up Period	Primary Outcome
Chae et al. [24]	2019	Republic of Korea	Single-center, retrospective	2012–2017	314	Age < 21 years, neuromuscular disease, impaired hepatic and renal function, combined or emergency procedures, non-malignant disease	314 (100)	Sugammadex vs. Pyridostigmine	30 days	Total and postoperative length of hospital stay; delayed discharge rate and readmission rate
Hunt et al. [25]	2020	USA	Single-center, retrospective	2014–2017	224	Age < 18 years, preoperative renal or hepatic failure, bowel obstruction, conversion laparotomy, postoperative mechanical ventilation, emergency surgery, ASA class > III, combination of sugammadex and neostigmine, glycopyrrolate use with sugammadex but without neostigmine, epidural anesthesia, bowel obstruction, open surgery, no documented postoperative bowel movement	224 (100)	Sugammadex vs. Neostigmine/Glycopyrrolate	In hospital	Time to first bowel movement (in hours) after reversal
Oh et al. [26]	2021	Republic of Korea	Single-center, retrospective	2014–2018	420	Robotic surgery, combined surgeries, non-malignant disease, direct postoperative ICU transfer, incomplete medical records, neuromuscular blockade other than rocuronium, deep neuromuscular blockade	420 (100)	Sugammadex vs. Pyridostigmine	In hospital	Postoperative respiratory adverse events
Serrano et al. [27]	2021	Spain	Multi-center, prospective (sub-study of POWER trial)	2017	676	Age < 18 years, emergency surgery, non-ERAS adherence	676 (100)	Sugammadex vs. Neostigmine	30 days	Moderate–severe postoperative complications, length of hospital stay
Traeger et al. [28]	2022	Australia	Single-center, retrospective	2019–2021	335	Age < 18 years, pelvic exenteration, robotic surgery, no reversal agent, combination of sugammadex and neostigmine, pyridostigmine prescription	335 (100)	Sugammadex vs. Neostigmine/Glycopyrrolate	30 days	Gastrointestinal recovery (GI-2): time of first bowel movement and tolerance of solid diet

**Table 2:** Patient characteristics.

Author	Reversal Agent	No. of Patients	Age (Years)	Sex (Male/Female)	BMI (kg/m^2^)	ASA Class (%)	Diabetes Mellitus (%)	Smoking History (%)	Cardiac Disease (%)	Pulmonary Disease (%)	Arterial Hypertension (%)
Chae et al. [24]	Sugammadex	157	62.5 ± 11.5 *	86/71	23.8 ± 3.3 *	ASA I 67 (43) ASA II 90 (57)	30 (19)	NS	76 (48)	6 (4)	NS
	Pyridostigmine	157	63.1 ± 11.8	83/74	23.4 ± 3.4	ASA I 77 (49) ASA II 80 (51)	26 (17)	NS	70 (45)	2 (1)	NS
Hunt et al. [25]	Sugammadex	96	60.68 (14.64) *	36/60	29.3 (6.09) *	ASA I-III 96 (100)	NS	16 (16.7)	NS	NS	NS
	Neostigmine/ Glycopyrrolate	128	60.34 (14.08)	58/70	29.6 (6.19)	ASA I-III 128 (100)	NS	36 (28.1)	NS	NS	NS
Oh et al. [26]	Sugammadex	210	68.0 [61.0;75.0] #	129/81	24.0 ± 3.3 *	ASA III-IV 32 (15.2)	NS	49 (23.3)	NS	27 (12.9)	NS
	Pyridostigmine	210	68.0 [60.0;74.0]	133/77	24.2 ± 3.4	ASA III-IV 28 (13.3)	NS	45 (21.4)	NS	21 (10.0)	NS
Serrano et al. [27]			All patients	All patients	All patients	All patients	All patients	All patients	All patients	All patients	All patients
	Sugammadex	563	67.9 (12.8) *	398/278	27.0 (4.7) *	ASA I 54 (8.0) ASA II 360 (53.3) ASA III 245 (36.2) ASA IV 17 (2.5)	141 (20.9)	126 (18.6)	108 (15.9)	104 (15.4)	348 (51.5)
	Neostigmine	113									
Traeger et al. [28]	Sugammadex	111	67 (57–76 [18–94]) †	62/49	28.7 (24.7–32.9 [18.2–73.0]) †	ASA I 3 (2.7) ASA II 41 (36.9) ASA III 62 (55.9) ASA IV 5 (4.5)	26 (23.4)	57 (51.4)	4 (3.6)	17 (15.3)	63 (56.8)
	Neostigmine/ Glycopyrrolate	224	64 (53–72 [19–90])	129/95	26.8 (23.4–30.4 [15.9–58.8])	ASA I 5 (2.2) ASA II 118 (52.7) ASA III 101 (45.1) ASA IV 0 (0)	39 (17.4)	112 (50)	7 (3.1)	15 (6.7)	93 (41.5)

BMI: body mass index, ASA: American Society of Anesthesiology, NS: not stated, * mean ± standard deviation, # median [IQR], † median (IQR [range]).

**Table 3:** Surgical and anesthesiologic features.

Author	Reversal Agent	NMBA	Anesthesia Time (min)	Laparoscopic Approach (%)	Cancer Surgery (%)
Chae et al. [24]	Sugammadex	Rocuronium	176.0 ± 46.7 *	32 (20)	157 (100)
	Pyridostigmine	Rocuronium	175.1 ± 41.0	34 (22)	157 (100)
Hunt et al. [25]	Sugammadex	Rocuronium or Vercuronium	229.8 (166.2) #	96 (100)	52 (54.2)
	Neostigmine/ Glycopyrrolate	Rocuronium or Vercuronium	214.2 (127.8)	128 (100)	67 (52.3)
Oh et al. [26]	Sugammadex	Rocuronium	202.5 [177.0; 240.0] #	210 (100)	210 (100)
	Pyridostigmine	Rocuronium	201.5 [170.0; 238.0]	210 (100)	210 (100)
Serrano et al. [27]		All patients	All patients	All patients	All patients
	Sugammadex	NS	NS	471 (69.7)	NS
	Neostigmine				
Traeger et al. [28]	Sugammadex	NS	170 (120–215 [29–443]) †	74 (66.7)	73 (65.8)
	Neostigmine/ Glycopyrrolate	NS	157 (110–194 [42–378])	111 (50.9)	123 (54.9)

NMBA: neuromuscular blocking agent, NS: not stated, * mean ± standard deviation, # median (IQR), † median (IQR [range]).

**Table 4:** Level of certainty of the evidence as assessed by the GRADE approach for the primary endpoint.

Outcomes	No. of Studies	No. of Included Patients		SMD/OR [95% CI]	Quality Assessment					Quality
		Sugammadex	Control		Risk of Bias ^a^	Inconsistency	Indirectness	Imprecision	Publication Bias	
Time to first postoperative bowel movement or flatus	2 [25,28]	207	352	SMD 13.01 [6.55–19.46]	Serious (−1)	Serious (−1)	No indirectness	No imprecision	NA	Very low

OR: odds ratio, SMD: standardized mean difference, NA: not available. ^a^ Risk of bias assessed using the ROBINS-I tool.

**Table 5:** Non-gastrointestinal motility outcomes.

					Heterogeneity Level	
Outcomes	No. of Included Studies	No. of Included Patients	SMD/OR [95% CI]	*p*-Value	I^2^ (%)	*p*-Value
Urinary tract infection	2 [24,27]	990	0.37 [0.07–2.04]	0.25	0	0.63
Pulmonary morbidity	3 [24,26,27]	1410	0.77 [0.46–1.29]	0.32	0	0.41
PONV	2 [25,26]	644	0.91 [0.59–1.41]	0.67	0	0.59
Length of postoperative hospital stay	4 [24,25,26,28]	1293	−0.03 [−0.27–0.21]	0.80	0	0.87
Anastomotic leak	2 [27,28]	970	1.11 [0.31–3.94]	0.87	61	0.11
Bleeding	2 [24,27]	990	0.76 [0.24–2.43]	0.64	3	0.31
Surgical site infection	2 [24,27]	990	0.65 [0.40–1.07]	0.09	0	0.74
PACU stay	2 [24,26]	734	−0.95 [−3.04–1.14]	0.37	0	0.91
Reoperation	3 [26,27,28]	1431	0.89 [0.54–1.48]	0.66	0	0.73
ICU readmission	2 [24,28]	649	1.16 [0.44–3.06]	0.76	32	0.23
Hospital readmission	3 [24,27,28]	1325	1.07 [0.68–1.67]	0.78	0	0.80
Mortality	2 [24,27]	990	1.89 [0.19–18.81]	0.59	61	0.11

ICU: intensive care unit, OR: odds ratio, PACU: postanesthesia care unit, PONV: postoperative nausea and vomiting, SMD: standardized mean difference.

## Data Availability

Not applicable.
